# Response to ALK‐TKIs in a lung adenocarcinoma patient harboring dual *DCTN1‐ALK* and *ALK‐CLIP4* rearrangements

**DOI:** 10.1111/1759-7714.14345

**Published:** 2022-02-24

**Authors:** Fangfang Gao, Fangfang Gao, Huijuan Wu, Junfeng Lu, Yaping Xu, Yanqiu Zhao

**Affiliations:** ^1^ Department of Internal Medicine Henan Cancer Hospital, Affiliated Cancer Hospital of Zhengzhou University Zhengzhou China; ^2^ Geneplus‐Beijing Beijing China

**Keywords:** ALK rearrangement, lung cancer, targeted therapy

## Abstract

Rearrangements involving anaplastic lymphoma kinase (*ALK*) gene have been reported in ~5% of non–small‐cell lung cancer patients. These rearrangements are characterized by the identification of various rare fusion partners, with unknown clinical significance. Specifically, the concurrence of different *ALK* fusions within the same patient, as well as its impact on therapeutic response to ALK tyrosine kinase inhibitors (ALK‐TKIs), are rarely reported. Here, we report a 46‐year‐old female who was diagnosed with lung adenocarcinoma and identified carrying concurrent *DCTN1‐ALK* and *ALK‐CLIP4* rearrangements by next generation sequencing (NGS) (638‐gene panel). This patient showed partial response to crizotinib with a progress‐free survival of 12 months and was then administered alectinib. Our report highlighted the importance of NGS testing in identifying rare ALK rearrangements and provided a novel insight into understanding the efficacy of ALK‐TKI in this subset of patients.

## Graphical abstract

Clinical case reports on ALK rearrangements are of vital significance for elucidating the association of targeted therapy efficacy with different ALK rearrangement types. This case report highlighted the importance of NGS testing in identifying rare ALK rearrangements, and provided a novel insight into understanding the efficacy of ALK‐TKI in this subset of patients.

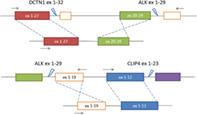

## INTRODUCTION

*ALK* rearrangements are reported in ~5% of non–small‐cell lung cancer (NSCLC) patients, and among them, *EML4‐ALK* is the most common fusion type.^1^ In addition to *EML4‐ALK*, many other rare fusion forms have been recognized from previous literatures and these non‐canonical fusions are usually considered unreliable predictors of matched targeted therapy efficacy.^2^ Moreover, concurrent multiple *ALK* rearrangements in a same patient are particularly rare because *ALK* rearrangements are well established driver events for NSCLC and a single alteration is considered sufficient to induce tumorigenesis. Here, we present the case of a 46‐year‐old NSCLC patient with concurrent *DCTN1‐ALK* (D27:A20) and *ALK‐CLIP4* (A19:C12) fusions, both of which are uncommon *ALK* fusion types. This patient received first‐line crizotinib treatment for 12 months with a best response of partial response (PR). Sequential administration of alectinib also showed PR and has lasted for at least 5 months.

### Case report

**FIGURE 1:**
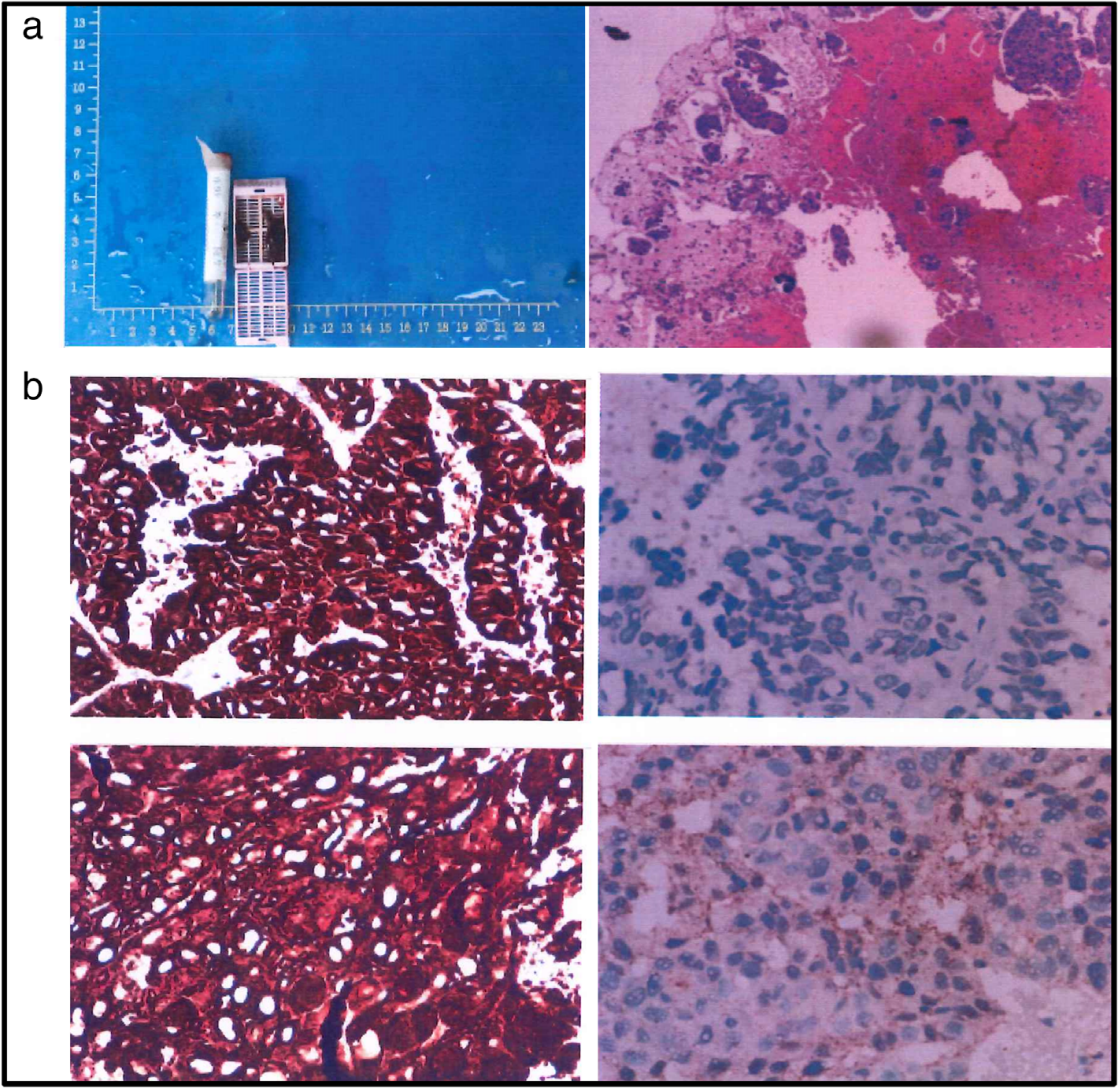
Immunohistochemistry (IHC) staining for ALK of lung tissue. (a) Integrative image of Formalin‐Fixed Paraffin‐Embedded (FFPE) tissue sample (left) and overview image of the sample using hematoxylin and eosin (HE) staining (right). (b) IHC staining for ALK. Strong positive staining for ALK in the left row and negative control staining in the right row

A 46‐year‐old non‐smoker female patient was diagnosed with lung cancer and admitted to our department in September 2019. A computer tomography (CT) scan showed an irregular mass with a diameter of 39 mm and unclear border in the upper lobe of the left lung and multiple enlarged lymph nodes in the left hilar, mediastinum, and bilateral subclavian. CT‐guided biopsy of the left lung mass was conducted and the pathological examination suggested that it was adenocarcinoma (Figure 1(a)). Immunohistochemical staining revealed positive ALK expression (Figure 1(b)) and negative PD‐1 or PD‐L1 expression (data not shown). The patient was finally diagnosed with stage IVA lung adenocarcinoma (T2N3M1) with a metastatic site of pleural dissemination (PL Dissem).

**FIGURE 2:**
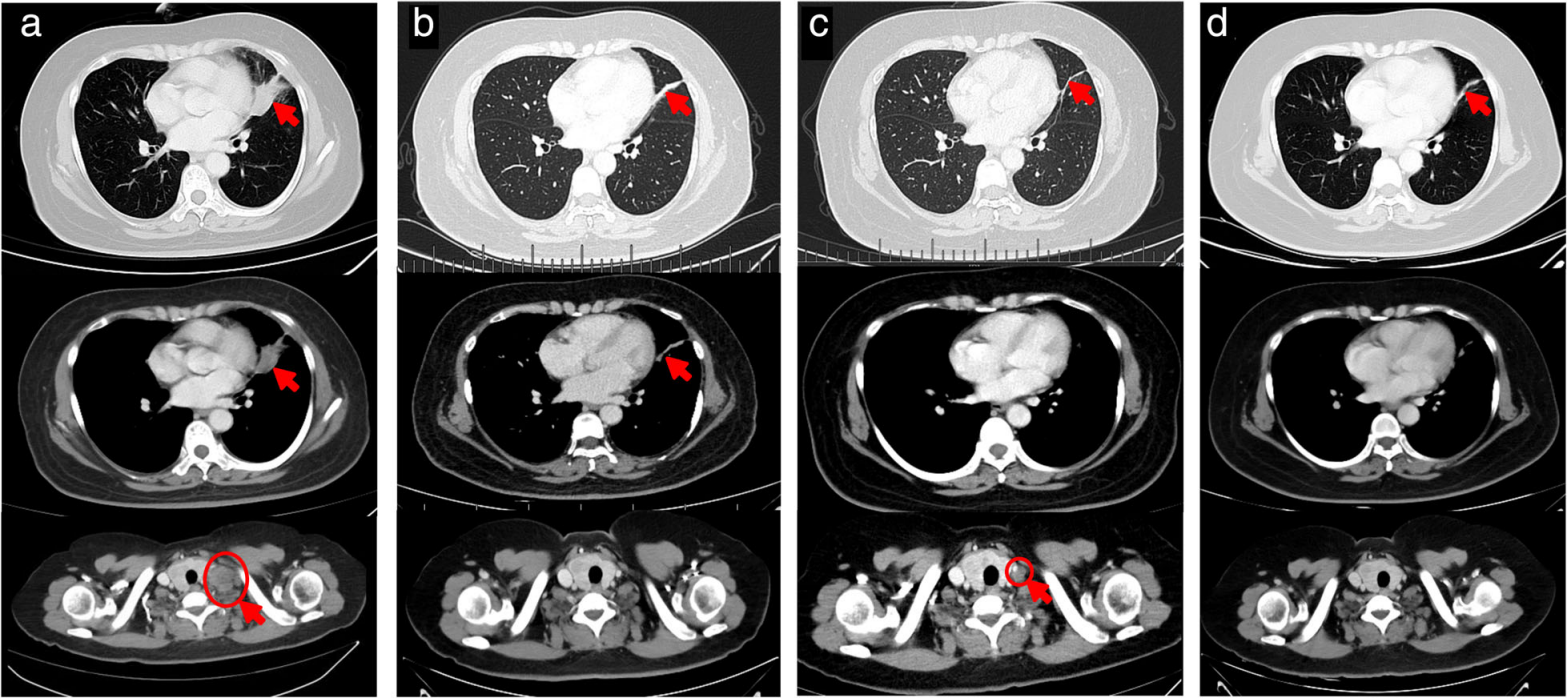
Subsequent chest CT scans. (a) Patient's CT scan at diagnosis, (b) 4 months after crizotinib treatment, (c) 12 months after initial crizotinib treatment, (d) 5 months after alectinib treatment. Red arrows indicate the tumor regions

Both tumor tissue and circulating tumor DNA (ctDNA) from this patient were sent for genetic testing by next‐generation sequencing (NGS) (638‐gene panel), and concurrent *DCTN1‐ALK* (D27:A20) and *ALK‐CLIP4* (A19:C12) rearrangements were identified in both tissue and ctDNA samples, with other concomitant mutations including *PIK3CB* L192R, *LRP1B* G4130R, and *TP53* S90P single nucleotide variants. The patient was treated with crizotinib (250 mg) twice daily since November 2019. After 4 months of therapy, the follow‐up CT scan revealed that the primary tumor was reduced to a diameter of 19 mm (Figure 2(a) and (b)), and the efficacy was evaluated as PR according to the Response Evaluation Criteria in Solid Tumors (RECIST1.1). One year later, the CT scan conducted in November 2020 showed that the metastatic lesions were enlarged although the primary site remained slightly reduced (Figure 2(c)), and the efficacy was assessed as progressive disease (PD). Alectinib was administered after an evaluation of patient's overall conditions. CT scan showed that a continuous response of at least 5 months was achieved with a best response of PR (Figure 2(d)).

## DISCUSSION

Several previous studies have reported that *DCTN1‐ALK* fusion could be detected in different types of tumors. Vendrell et al.^3^ found that among a large cohort encompassing 1128 NSCLC patients, only one patient was detected with *DCTN1‐ALK* fusion, and a 3‐month treatment of crizotinib displayed efficacy with tumor shrinking significantly in this patient. Another study reported an inflammatory myofibroblastoma case who harbored *DCTN1‐ALK* fusion and received crizotinib for more than 6 months with efficacy evaluated as partial remission.^4^ However, because of the rareness of *DCTN1‐ALK* fusion in NSCLC patients, the efficacy and duration of ALK‐TKI treatment in this subset of patients remained poorly understood. Here, we identified a female patient in our center with concurrent *DCTN1‐ALK* (D27:A20) and *ALK‐CLIP4* (A19:C12) fusions and reported that this patient achieved PR to first‐line crizotinib therapy with a progress‐free survival (PFS) of 12 months, which was very similar to that of patients carrying *EML4‐ALK* fusion.^5^ This may be because of the fact, that like *EML4*, the *DCTN1* fragment also contains a functional region that can initiate *ALK* transcription and translation.^4^

**FIGURE 3:**
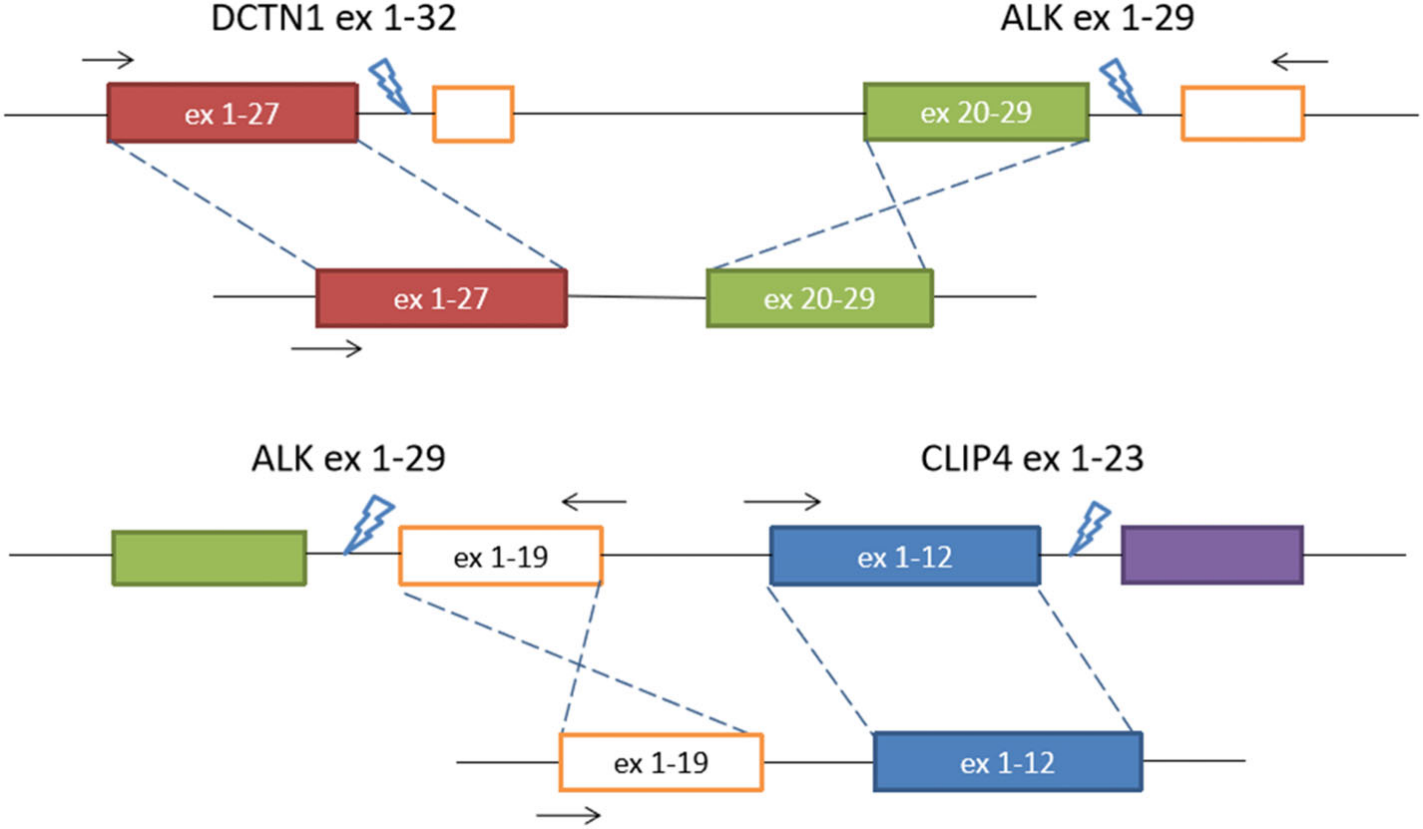
Schematic illustration of DCTN1‐ALK and ALK‐CLIP4 gene rearrangement

It is worth noting that *ALK‐CLIP4* (A19:C12) rearrangement (Figure 3) was detected in both plasma and tumor tissue before crizotinib treatment. This fusion form lacked the region coding for ALK kinase domain, which was located downstream of exon 20.^6^ Whether the co‐occurred *ALK‐CLIP4* fusion impacts ALK‐TKI efficacy needs more studies to elucidate. However, considering that *ALK‐CLIP4* fusion may not lead to the expression of the functional ALK protein, we speculated that this fusion type would not respond to ALK‐TKI treatment.

In conclusion, this study reported an ALK‐rearranged NSCLC case with dual ALK fusions and who responded to crizotinib treatment with a duration time of approximately 1 year. Sequential alectinib administration also proved to be effective for this patient. This case provided novel clues facilitating systematic treatments for other similar cases. Future work including genetic monitoring via liquid biopsy and the functional validation of the *ALK* fusions mentioned above is urged. Our case reports the efficacy of ALK‐TKIs and is conducive to clinical decision making on patients with rare driver mutations.

## CONFLICT OF INTEREST

The authors declare no conflicts of interest.
